# Red Flags for early referral of people with symptoms suggestive of narcolepsy: a report from a national multidisciplinary panel

**DOI:** 10.1007/s10072-018-3666-x

**Published:** 2018-12-12

**Authors:** L. Vignatelli, E. Antelmi, I. Ceretelli, M. Bellini, C. Carta, P. Cortelli, L. Ferini-Strambi, R. Ferri, R. Guerrini, F. Ingravallo, V. Marchiani, F. Mari, G. Pieroni, F. Pizza, M. C. Verga, E. Verrillo, D. Taruscio, Giuseppe Plazzi

**Affiliations:** 1 IRCCS Istituto delle Scienze Neurologiche di Bologna, Bologna, Italy; 2 Department of Biomedical and Neuromotor Sciences, University of Bologna, via Ugo Foscolo n 7, 40123 Bologna, Italy; 3 Associazione Italiana Narcolettici e Ipersonni (AIN), Florence, Italy; 4 Azienda USL Toscana centro Sedi di Prato, Prato, Italy; 5 National Centre for Rare Diseases, Istituto Superiore di Sanità, Rome, Italy; 6 Department of Clinical Neurosciences, Neurology - Sleep Disorders Center, IRCCS San Raffaele Scientific Institute, Milan, Italy; 7 Sleep Research Centre, Department of Neurology I.C., Oasi Research Institute – IRCCS, Troina, Italy; 8 Pediatric Neurology, Neurogenetics and Neurobiology Unit and Laboratories, Children’s Hospital A. Meyer-University of Florence, Florence, Italy; 9 Department of Medical and Surgical Sciences (DIMEC), University of Bologna, Bologna, Italy; 10 Child Neuropsychiatric Unit, Polyclinic S. Orsola-Malpighi, University of Bologna, Bologna, Italy; 11 Primary Care Pediatrics, ASL Salerno, Vietri sul Mare, SA Italy; 12 Sleep and Long Term Ventilation Unit, Pediatric Pulmonology & Respiratory Intermediate Care Unit, Academic Department of Pediatrics (DPUO) Bambino Gesù Children’s Hospital, IRCCS, Rome, Italy

**Keywords:** Narcolepsy, Cataplexy, Diagnostic delay, Red flags, Diagnostic criteria, Burden of disease

## Abstract

**Objective:**

Narcolepsy is a lifelong disease, manifesting with excessive daytime sleepiness and cataplexy, arising between childhood and young adulthood. The diagnosis is typically made after a long delay that burdens the disease severity. The aim of the project, promoted by the “Associazione Italiana Narcolettici e Ipersonni” is to develop *Red Flags* to detect symptoms for early referral, targeting non-sleep medicine specialists, general practitioners, and pediatricians.

**Materials and methods:**

A multidisciplinary panel, including patients, public institutions, and representatives of national scientific societies of specialties possibly involved in the diagnostic process of suspected narcolepsy, was convened. The project was accomplished in three phases. Phase 1: Sleep experts shaped clinical pictures of narcolepsy in pediatric and adult patients. On the basis of these pictures, *Red Flags* were drafted. Phase 2: Representatives of the scientific societies and patients filled in a form to identify barriers to the diagnosis of narcolepsy. Phase 3: The panel produced suggestions for the implementation of *Red Flags*.

**Results:**

*Red Flags* were produced representing three clinical pictures of narcolepsy in pediatric patients ((1) usual sleep symptoms, (2) unusual sleep symptoms, (3) endocrinological signs) and two in adult patients ((1) usual sleep symptoms, (2) unusual sleep symptoms). Inadequate knowledge of symptoms at onset by medical doctors turned out to be the main reported barrier to diagnosis.

**Conclusions:**

This report will hopefully enhance knowledge and awareness of narcolepsy among non-specialists in sleep medicine in order to reduce the diagnostic delay that burdens patients in Italy. Similar initiatives could be promoted across Europe.

**Electronic supplementary material:**

The online version of this article (10.1007/s10072-018-3666-x) contains supplementary material, which is available to authorized users.

## Introduction

Narcolepsy is characterized by the pentad of excessive daytime sleepiness, cataplexy, hypnagogic or hypnopompic hallucinations, sleep paralysis, and disrupted nighttime sleep [1–3]. It is a chronic and disabling disorder, with a bimodal peak for age at onset, with a maximal rate of onset at 15 years and the second peak at 35 years, needing a lifelong treatment [4].

Narcolepsy has a prevalence between 20 and 50 per 100,000 [5–11] and is recognized as rare disease (RF 0150) by the Italian Ministry of Health [attachment 1 at DM N. 279/2001]. At December 31, 2014, 610 patients were recorded in Italy by the National Registry for Rare Diseases [12], with an average national annual incidence in the 3-year period 2012–2014 of 0.97 per 1 million person-years [13] compared to an annual incidence of 1.37 per 100,000 person-years in Olmsted County, Minnesota (USA) [7].

The current classification [1] distinguishes narcolepsy type 1 (NT1) and narcolepsy type 2 (NT2). Both types feature daytime sleepiness confirmed by polysomnographic studies, while hypocretin level of less than 110 pg/mL and the presence of cataplexy characterize only NT1. Therefore, NT1 is linked to the loss of hypothalamic hypocretinergic neurons, while the cause of NT2 remains unknown. Both genetic and environmental factors play a crucial role in the pathogenesis of narcolepsy. Most patients with narcolepsy carry HLA-DQB1*0602 and a link to polymorphisms in other non-HLA genes that may affect immune regulatory function and to several infectious triggers, supporting an autoimmune pathogenesis [2, 3].

Although narcolepsy has an early onset, a diagnostic delay that often exceeds 10 years from symptoms onset is reported [14–18]. Lately, there has been a trend toward a shorter diagnostic delay, which could potentially be attributed to an increased awareness of narcolepsy, as for example in Italy through media campaigns [https://www.narcolessia.org]. However, as emerged from a recent survey conducted by the Italian association of patients with narcolepsy and hypersomnia (Associazione Italiana Narcolettici e Ipersonni – AIN; https://www.narcolessia.org), diagnostic delay continues to be an issue, which makes identifying its causes a priority.

Data from several studies and reviews suggest that the primary reason for diagnostic delay is likely the lack of symptoms recognition, often resulting in misdiagnosis [14–16, 18–22]. This may originate from several factors, including lack of recognition, on the part of the clinician, of the signs and symptoms of narcolepsy, leading to multiple referrals before receiving a proper diagnosis [14, 20–22] as well as misdiagnosis of narcolepsy as another condition, such as epilepsy, depression, or attention-deficit/hyperactivity disorder, which further delays treatment [14, 19, 20, 23, 24].

Receiving a correct narcolepsy diagnosis is especially relevant in children, in order to timely face consequences, such as low academic achievement and social difficulties [18]. This is an important pitfall to be addressed, as misdiagnosis is more common in pediatric narcolepsy [19, 20, 25]. Indeed, the strongest predictor of a delayed diagnosis, along with the absence of cataplexy, has been reported to be pediatric onset of symptoms [26]. Diagnosis of narcolepsy indeed may be particularly challenging in children as symptoms may manifest differently from adults. Excessive daytime sleepiness can manifest as attentional problems, paradoxical hyperactivity, elongation of nighttime sleep, and resuming of postprandial sleep [3]. Hallucinations can be underrecognized because of the difficulty for children to recognize and describe them and for the possible coexistence of nightmares, rapid eye movement (REM) sleep behavior disorder (RBD), sleep terrors, and confusional arousals [26, 27]. Children with NT1 may have peculiar features of cataplexy with subcontinuous hypotonia, mainly involving the facial district “cataplectic face” and superimposed dyskinesia [23, 24, 28].

Timely diagnosis means better outcomes and it has been reported that patients diagnosed before age 30 years exhibit lower unemployment rates and have better health perception than those diagnosed at a later age [29], therefore resulting in reduced psychological distress along with socioeconomic and healthcare consequences [30–34]. Accordingly, it is important to understand the reason of this pitfall in diagnosis and to act in order to correct this gap and improve the rate of a timely diagnosis.

Considering these premises and the result of the above-mentioned AIN survey, AIN itself has promoted this document with the specific aim of exploring, at multiple levels of the healthcare system, the causes of diagnostic pitfalls and of proposing corrective actions. We propose of doing this through *Red Flags* aimed at detecting narcolepsy symptoms early and timely referring potentially affected individuals to specialist care.

## Objective

The aim of the project is to develop *Red Flags* to improve the knowledge about the symptoms suggestive of narcolepsy among non-sleep medicine expert physicians potentially involved in the first referral of patients (neurologists, pediatric neurologists, general practitioners, and pediatricians) for early referral to sleep medicine centers. The focus is the definition of the exemplary (*chief complaint*) and atypical (*variant complaint*) clinical presentations of narcolepsy, at its onset or when fully developed, in children and in adults. The analysis of barriers and facilitators of the diagnostic suspicion of narcolepsy was carried out with the aim of suggesting strategies for the implementation of the *Red Flags*.

## Methods

The current document is a position paper, promoted by AIN, based on experts’ and stakeholders’ opinions. The following scientific societies endorsed the project by appointing a representative member of each society: the Italian society of neurology (Società Italiana di Neurologia, SIN; P. C.); the Italian society of pediatric neurology (Società Italiana Neurologia Pediatrica, SINP; V. M.); the Italian society of social and preventive medicine (Società Italiana di Pediatria Preventiva e Sociale, SIPPS; M.C. V.); the Italian society of pediatrics (Società Italiana di Pediatria, SIP; E. V.); the Italian society of general practitioners (Società Italiana di Medicina Generale, SIMG; M. B.); the Italian society of neurophychiatrists for children and adolescents (Società italiana di neuropsichiatria dell’infanzia e dell’adolescenza, SIMPIA; R. G., F. M.); the World Association of Sleep Medicine (WASM; L. F-S.); the Italian association of sleep medicine (Associazione Italiana Medicina del Sonno, AIMS; R. F.), the Italian association of healthcare managers (Associazione Nazionale Medici di Direzione Ospedaliera; ANMDO; G. P.). A scientific committee was established, including a patients’ representative (I. C., AIN), methodology experts from IRCCS Istituto delle Neuroscienze di Bologna (L. V.) and Italian institute of health - National Centre for Rare Diseases (Istituto Superiore di Sanità, ISS: C. C., D. T.), and narcolepsy experts (E. A., F. P., F. I., L. F-S., R. F. and G. P.).

The document was planned at an initial meeting of the scientific committee and scientific societies spokespersons in Bologna (Italy), July 21, 2017. Three phases were conceived: (1) *Red Flags* editing; (2) barriers and facilitators analysis; (3) suggestions for *Red Flags* implementation*.*

### Red Flags editing

Based on the AASM ICSD 3 [1] criteria for narcolepsy and by consulting evidence-based summaries [35–37], sleep experts defined the exemplary and atypical clinical pictures of narcolepsy, at onset or fully developed, in children and in adults. In particular, each *Red Flag* was edited as a short statement including the alerting symptom(s), either according to the possible chief complaints or the possible variant complaints. An explanation box, reporting examples of real-life presentation of symptoms, equipped each *Red Flag*. For each piece of evidence at the basis of the statement, the possible references labeled with their study design were reported. A first draft was submitted to the whole members group and a final consensus version was achieved. *Red Flags* were edited in Italian then translated into English (See Supplementary Material).

### Barriers and facilitators analysis

An electronic questionnaire was built to investigate barriers and facilitators of the diagnostic suspicion of narcolepsy. The questionnaire was designed according to the “Tailored Implementation for Chronic Diseases” (TICD) checklist [38], a comprehensive tool that identifies determinants, that is barriers and facilitators, of healthcare professional practice. Briefly, it includes 57 potential determinants of practice, grouped in seven domains (guideline factors; individual health professional factors; patient factors; professional interactions; incentives and resources; capacity for organizational change; and social, political, and legal factors). The scientific committee chose the relevant domains and determinants conceivably involved in the diagnostic process of patients with suspicion of narcolepsy. Each determinant was transformed as question with a 4-point Likert scale (possible answers: “strongly agree,” “agree,” “disagree,” “strongly disagree”). The checklist was then edited in three versions and submitted to the scientific societies spokespersons, child patient caregivers, and adult patients. Patients and caregivers were invited by AIN.

### Suggestions for Red Flags implementation

A list of suggestions aiming at the implementation of the *Red Flags*, based on the results of the Phase 2 analysis, was edited by the whole group during a final meeting.

## Results

*Red Flags* were produced representing three clinical pictures of narcolepsy in children (picture with (1) usual sleep symptoms, (2) unusual sleep symptoms, (3) metabolic symptoms) and two in adults (picture with (1) usual sleep symptoms, (2) unusual sleep symptoms). See Supplementary Material for original version in Italian.

### Red flags for pediatric patients

Narcolepsy should be suspected in pediatric patients that present with…

1. **Excessive daytime sleepiness and/or cataplexy** (see Tables 1 and Table 2 for explanations).

**Table 1:** Explanation of pediatric patients *Red Flag 1* on excessive daytime sleepiness. This *Red Flag* was formulated based on core literature consisting of the international diagnostic criteria [1], one evidence-based clinical summary [36], three reviews [3, 39, 40], one cohort study [24], one incidence cross-sectional study [41], one case report [42]

In pediatric patients **excessive daytime sleepiness** may present with:	
• … **sleep attacks**, occurring in unusual and non-monotonous conditions, usually of brief duration, with a refreshing effect and with the recall of vivid dreams	
• .. **change (acute or progressive) of sleep-wake circadian rhythm** with the tendency to increase the total sleep hours per day, to resume the habitual napping that occurs beyond the age when a child typically grows out of the need for daily naps (i.e. after five to six years of age), to go to sleep earlier and to wake up later and with major difficulties in the morning awakening	
• … onset during daytime of **hyperactivity, inattention, irritability and automatic behaviors**. Those symptoms are more easily observable at school, as for example the child progressively starts writing with a shoddy handwriting or with incomprehensible spelling until interrupting completely the handwriting	
*It should be kept in mind that in children the most frequent differential diagnoses of these symptoms are chronic sleep deprivation, psychological problems, seizures, infectious encephalopathies. Therefore, if one or more of these conditions are suspected, narcolepsy should also be considered in the differential diagnosis.*

**Table 2:** Explanation of pediatric patients *Red Flag 1* on cataplexy. This *Red Flag* was formulated based on core literature consisting of the international diagnostic criteria [1], one evidence-based clinical summary [36], one review [3], one cohort study [24], two case-series [23, 28], two case reports [43, 44]

In pediatric patients **cataplexy** may present with:	
• … brief (seconds-minutes) episodes of **loss of muscle tone** triggered by emotions, during wakefulness. The loss of muscle tone can be partial (eyelid ptosis, mouth opening, tongue protrusion, head drop, slurred speech, trunk drop) and/or generalized (up to fall to the ground)	
• … **cataplectic face, droopy expression,** with the nearly constant but fluctuating presence of eyelid ptosis, mouth opening, tongue protrusion. Cataplectic face may be present sub-continuously with intermittent exacerbation due to daily life activities (i.e. eating, playing or being involved in an activity with emotional involvement, as playing with videogames, watching a funny movie, etc.)	
• … **floppy aspect**, that may involve mainly the head and the trunk (with intermittent episodes of head and trunk drops) or all the body and the legs appearing limply with a wobbly, wide-based, unstable, ataxic-like gate	
• **intermittent active movements**, mainly involving the face (grimacing, eyebrow raising, perioral dyskinesias, tongue thrusting movements, tongue protrusion) or the body (stereotypies and choreic-like movements)	
*It should be kept in mind that in pediatric patients the most frequent differential diagnoses of these symptoms are neuromuscular disorders and hyperkinetic movement disorders. Therefore, if such conditions are suspected, narcolepsy should also be considered in the differential diagnosis. The fluctuating character (not triggered by exercise) and the association with excessive daytime sleepiness may help in differential diagnosis.*	

➔*In this case, it is recommended that the patient should be referred to the reference centers for the diagnosis.*

2. peculiar **sleep-related disorders**, **i.e., hypnagogic-hypnopompic hallucination, sleep paralysis, and impaired nocturnal sleep** (see Table 3 for explanations).

**Table 3:** Explanation of pediatric patients *Red Flag 2* on peculiar sleep-related disorders. This *Red Flag* was formulated based on core literature consisting of the international diagnostic criteria [1], one evidence-based clinical summary [36], two reviews [3, 45], four case-series [46–49]

In pediatric patients **peculiar sleep-related disorders** may present with:	
• Multimodal **hallucinations** (mainly visual) or illusions that appear at sleep onset (hypnagogic) or on awakening (hypnopompic)	
• **Vivid dreams**, mainly with frightening contents	
• **Sleep paralyses**, i.e. inability to move for one or two minutes immediately after awakening or just before falling asleep, accompanied by a disquieting sensation with or without concomitant hallucinations/illusions	
• **Impaired nocturnal sleep**, with agitations, fragmented sleep and **REM sleep behavior disorder (RBD),** i.e. with the presence of movements that mimic the dream content (enacted dreams) (i.e. the patient moves as if he/she were acting what he/she is dreaming; upon awakening he/she recalls a vivid dream coherent with the performed movements)	
*It should be kept in mind that in pediatric patients in the full-blown disorder, these symptoms are usually accompanied by excessive daytime sleepiness and cataplexy. It is rare, but possible, that these symptoms represent the initial manifestations of the disease. In children, given the low insight and the embarrassment in reporting unpleasant or embarrassing experiences, these are rarely reported spontaneously and difficult to investigate.*	

*➔ In this case, it is recommended that the presence of excessive daytime sleepiness and cataplexy (as indicated in Red Flag 1) should be investigated, even if these symptoms are not directly reported by the patient. In case of positive history of excessive daytime sleepiness and/or cataplexy, it is recommended that the patient should be referred to the reference centers for the diagnosis*

3. signs of **precocious puberty** and/or increase of weight until the development of **obesity** (see Table 4 for explanations)**.**

➔ *In this case, it is recommended that the presence of excessive daytime sleepiness and cataplexy (as indicated in Red Flag 1) should be investigated, even if these symptoms are not directly reported by the patient. In case of positive history of excessive daytime sleepiness and/or cataplexy, it is recommended that the patient should be referred to the reference centers for the diagnosis.*

### Red flags for adult patients

Narcolepsy should be suspected in adult patients that present with …

1. **Excessive daytime sleepiness and/or cataplexy** (see Tables 5 and 6 for explanations).

**Table 4:** Explanation pediatric patients Red Flag 3 on endocrinological signs. This *Red Flag* was formulated based on core literature consisting of the international diagnostic criteria [1], one evidence-based clinical summary [36], one review [3], one cohort study with controls [50], one retrospective cohort study [51], one cross-sectional study with controls, [52], one case-series [53]

In children, near to the onset, narcolepsy can be accompanied by **endocrinological signs**, i.e. acceleration/anticipation of pubertal signs until reaching criteria for precocious puberty and the progressive increase of weight until reaching the criteria for obesity. In these conditions therefore, it is recommended that the symptoms of narcolepsy should be investigated as reported in *Red Flags* 1 e 2.

**Table 5:** Explanation of adult patients *Red Flag 1* on excessive daytime sleepiness. Red Flag 1 on excessive daytime sleepiness was formulated based on core literature consisting of the international diagnostic criteria [1], two evidence-based clinical summaries [35, 37], one review [2]

In adult patients **excessive daytime sleepiness** may present with:	
• … **sleep attacks**, occurring in unusual and non-monotonous conditions, usually of brief duration, with a refreshing effect and with the recall of vivid dreams	
•... **change (acute or progressive) of sleep-wake circadian rhythm** with the tendency to increase the total sleep hours per day and to be prone to fall asleep throughout the day, often at inappropriate times	
*It should be kept in mind that in adults the most frequent differential diagnoses for these symptoms are obstructive sleep apnea, chronic sleep deprivation, psychological problems, and seizures. Therefore, if one or more of these conditions are suspected, narcolepsy should also be considered in the differential diagnosis.*

**Table 6:** Explanation of adult patients *Red Flag 1* on cataplexy. This *Red Flag* was formulated based on core literature consisting of the international diagnostic criteria [1], two evidence-based clinical summaries [35, 37], one review [2], three case-series [54–56].

In adult patients **cataplexy** may present with:	
• … brief (seconds-minutes) episodes of **loss of muscle tone** triggered by emotions, during wakefulness. The loss of muscle tone can be partial (eyelid ptosis, mouth opening, tongue protrusion, head drop, slurred speech, trunk drop) and/or generalized (up to fall to the ground)	
• … transient episodes (seconds-minutes) with **speech and action arrest** without a clear hypotonia, but always with retained consciousness.	
*It should be kept in mind that in adult patients the most frequent differential diagnoses of these symptoms are seizures and cerebrovascular attacks. Therefore, if such conditions are suspected, narcolepsy should also be considered in the differential diagnosis. The absence of an aura and the integrity of consciousness in narcolepsy may help in the differential diagnosis.*	

➔*In this case, it is recommended that the patient should be referred to the reference centers for the diagnosis.*

2. peculiar **sleep-related disorder**, **i.e., hypnagogic-hypnopompic hallucination, sleep paralysis, and impaired nocturnal** (see Table 7 for explanations).

**Table 7:** Explanation of adult patients *Red Flag 2* on peculiar sleep-related disorders. This *Red Flag* was formulated based on core literature consisting of the international diagnostic criteria [1], two evidence-based clinical summaries [35, 37], three reviews [2, 57, 58], two case-series [59, 60]

In adult patients **peculiar sleep-related disorders** may present with:	
• Multimodal **hallucinations** (mainly visual) or illusions which present at sleep onset (hypnagogic) or on awakening (hypnopompic)	
• **Sleep paralyses**, i.e. inability to move for one or two minutes immediately after awakening or just before falling asleep, accompanied by a disquieting sensation with or without concomitant hallucinations/illusions	
• **Impaired nocturnal sleep**, with agitations, fragmented sleep and **REM sleep behavior disorder (RBD),** i.e. with the presence of movements that mimic the dream content (enacted dreams) (i.e. the patient moves as if he/she were acting what he/she is dreaming; upon awakening he/she recalls a vivid dream coherent with the performed movements)	
*It should be kept in mind that in adult patients these symptoms may occur as isolated phenomena, such as for example isolated idiopathic RBD or RBD in neurodegenerative conditions. However, in presence of one or more of these conditions, the co-occurrence of excessive daytime sleepiness and of cataplexy should also be investigated.*	

*➔ In this case, it is recommended that the presence of excessive daytime sleepiness and cataplexy (as indicated in Red Flag 1) should be investigated, even if these symptoms are not directly reported by the patient. In case of positive history of excessive daytime sleepiness and/or cataplexy, it is recommended that the patient should be referred to the reference centers for the diagnosis.*

### Barriers and facilitators analysis

The following domains and determinants were selected from the TICD checklist to design and to analyze questionnaire results: (a) individual health professional factors (knowledge and skills; cognition and attitudes; professional behavior); (b) patient factors (needs; beliefs and knowledge; preferences; motivation; behavior); (c) professional interactions (referral processes); (d) incentives and resources (nonfinancial incentives and disincentives; assistance for clinicians); (e) capacity for organizational change (regulations, rules policies); (f) social, political, and legal factors (legislation).

#### Scientific societies spokespersons

Five specialist spokespersons completed the survey (a general practitioner, a neurologist, a child neuropsychiatrist, a child neurologist, a general pediatrician).

For most specialists, main barriers of diagnostic suspicion of narcolepsy are the insufficient knowledge of symptoms at onset, and the insufficient skill or propensity to examine symptoms when they are considered atypical (elongation of 24-h sleep, hallucinations, cataplexy, nighttime sleep disruption with rapid eye movement (REM) sleep behavior disorder, sleep terrors) or when they are reported as disease consequences (attentional problems, paradoxical hyperactivity, worsening of school or work performances). Another barrier is represented by the difficult referral process to expert sleep centers, due to the geographical distance or the difficulty of booking an appointment. Social or legal factors are not deemed as a relevant cause of the diagnostic delay; however, the fear of losing the driving license was raised by one specialist as a possible diagnostic barrier. Possible facilitators are tools to elicit the diagnostic suspicion or to improve the knowledge (questionnaires and educational materials, both printed and on the web; toll-free number) and organizational changes to simplify the access to expert sleep centers (dedicated outpatients’ clinic; diagnostic–therapeutic pathway).

#### Pediatric patients

Twenty-three caregivers of pediatric patients (14 males/9 females) completed the survey; narcolepsy type 1 was the prevalent diagnosis (21). The sample was quite homogeneously distributed in the Italian areas (north west 39%; north east 14%; middle 30%; south and islands 17%). Median age at onset was 9 years, and mean diagnostic delay 2.1 years (SD 3.5; median 1; range 0–14). Narcolepsy was not suspected at the first visit in 61% of patients; 47% of them consulted more than two physicians, and up to 10 in one case. Reported alternative diagnoses (in 48% of patients) are other organic disorders (epilepsy, obstructive sleep apnea syndrome, brain neoplasm, iron deficiency, drug abuse, tics, precocious puberty), psychological problems, abnormal attitudes/behaviors (boredom, laziness).

Main barrier to the diagnostic suspicion of narcolepsy was the inadequate knowledge of the disease by physicians (see Table 8). Relevant was also the scarce awareness of symptoms in their child by parents. To be noticed, the presence of more popular competing disorders (see above) among the possible barriers to diagnostic suspicion.

**Table 8:** Main barriers of diagnostic suspicion of narcolepsy according to caregivers of pediatric patients and adult patients

Determinants (domain and specific determinant)	Judged as relevant (% out of 23 pediatric patients’ caregivers)	Judged as relevant (% out of 48 adult patients)
Patient factors (beliefs and knowledge; behavior)		
Scarce awareness of symptoms by parents/patients	61%	64%
Individual health professional factors (knowledge and skills)		
Insufficient knowledge of the disease	79%	69%
Professional interactions (referral processes)		
Difficult access to expert sleep center	26%	23%
Social, political, and legal factors		
Social factors or legislation as barriers of the healthcare process	4%	34%
Barriers of diagnostic suspicion (open question)		
Insufficient knowledge of the disease by the physician	46%	55%
No awareness of symptoms by parents/patients	13%	12%
More popular competing disorders	8%	11%
Prejudices/social barriers	8%	8%
No knowledge of specialist centers	–	2%
None/no response	25%	12%

#### Adult patients

Forty-eight adult patients (25 males/23 females) completed the survey; narcolepsy type 1 was the prevalent diagnosis (32). The sample was quite homogeneously distributed in the Italian areas (north west 26%; north east 22%; middle 22%; south and islands 30%). Median age at onset was 11 years, mean diagnostic delay 6.7 years (SD 7.3; median 3.5; range 0–42). Narcolepsy was not suspected at first visit in 67% of patients; 59% of them consulted more than two physicians, and up to 10 in three cases. Reported alternative diagnoses (in 48% of patients) included other organic disorders (42%: epilepsy, obstructive sleep apnea syndrome, restless leg syndrome, gastroesophageal reflux, post-viral syndrome, obesity) and psychiatric disorders (43%: depression, anxiety), abnormal attitudes/behaviors (15%: laziness, tiredness).

Main barrier to the diagnostic suspicion of narcolepsy is the insufficient knowledge of the disease by physicians (see Table 8) and the scarce awareness of symptoms by patients. To be noticed, the presence of more popular competing disorders among the possible barriers to diagnostic suspicion. The presence of social/legislation factors (not acceptance of the disease, fear of social and work limitations, fear of driving license loss) is also reported as relevant by 1/3 of patients.

### Suggestions for Red Flags implementation

After the analysis of the above reported data, the multidisciplinary panel agreed on the following suggestions to implement the above reported *Red Flags*.

- The *Red Flags* should be the object of a national campaign, with presentation at national congresses and meetings and publications in Italian journals targeted to the specialties relevant to the project.
- The *Red Flags* should be disseminated with simple educational materials, such as leaflets with cartoons or as web movies that can be distributed in primary care centers, or at school, where symptoms can be firstly recognized.
- To facilitate the access to the reference centers, in collaboration with the National Helpline on Rare Diseases (Telefono Verde Malattie Rare 800 89 69 49) of the Istituto Superiore di Sanità (National Centre for Rare Diseases), a national central call-center could be designed in order to drive and help clinicians in referring the patients to the nearest and more appropriate center.
- The recognition of the symptoms by physicians could be elicited by validated questionnaires. Due to the scarcity of these tools in the Italian language (e.g., [61, 62] considering only excessive daytime sleepiness), a tool including the whole *Red Flags* should be devised and validated.
- The efficacy of the *Red Flags* implementation in reducing the diagnostic gap of patients with narcolepsy could be the object of a national multi-center study (e.g., an interrupted time series study).
- Similar initiatives could be promoted across European countries.

## Discussion

Narcolepsy is still a disease burdened by an unacceptable diagnostic delay [18, 26] and possibly by missed diagnoses in Italy [12, 13].

The persistence of a substantial delay in recognizing symptoms of narcolepsy, even among sleep medicine specialists, has been highlighted also by the Awareness and Knowledge of Narcolepsy (AWAKEN) study [21]. This is a survey including a sample of 1000 US adults, 300 primary care physicians (PCPs), and 100 sleep medicine specialists conducted in the USA. The study showed that even if 62% of sleep specialists and 24% of PCPs considered themselves “very” or “extremely” knowledgeable about narcolepsy, only 42% and 9% of sleep specialists and PCPs, respectively, felt “very” or “extremely” comfortable diagnosing the disorder. The striking finding was however that only 22% of sleep specialists and 7% of PCPs identified all 5 key narcolepsy symptoms; particularly, while excessive daytime sleepiness and cataplexy were more easily recognized, the other symptoms were instead more rarely investigated and recognized.

An Italian survey disclosed that pediatricians and child neuropsychiatrists scored low in all areas of sleep knowledge and particularly in questions about narcolepsy, sleep apnea, and parasomnias [63].

In order to reach patients’ needs and overcome the diagnostic gap, AIN has promoted the initiative at issue aiming at analyzing the reasons that hamper a timely diagnosis and to offer to general clinicians, pediatricians, and neurologists a document containing *Red Flags* that can help in a prompt recognition of symptoms that can lead to suspect narcolepsy, in order to refer the patient to the reference centers for the diagnosis.

As far as barriers analysis is concerned, results of questionnaires confirmed that both patients and clinicians judged lack of knowledge of symptoms as a main factor hampering a right and timely diagnosis. Particularly, as emerged also in the AWAKEN study [21], the scarce knowledge about symptoms different from excessive daytime sleepiness and cataplexy emerges as one of the main causes in delaying the proper recognition of the disease.

Indeed, in more than 60% of both pediatric and adult patients, narcolepsy was not suspected at the first outpatient evaluation; and therefore nearly 60% of patients needed to consult more than two physicians. In over 50% of cases, a diagnosis of different organic conditions (epilepsy, sleep apnea, brain lesions) or of a psychiatric condition was made.

Noteworthy, what came to light from our survey was the shorter mean diagnostic delay in Italy compared to other European data (namely 2.1 years for pediatric cases and 6.7 years for adults). This could be due to the shortening of the delay in the last years (note the difference between children and adults), also thanks to awareness campaigns on narcolepsy that AIN has been promoting since 2000. These data could also be biased by the small sample size and the recruitment process (voluntary).

Considering the differences in phenotypic presentations in adults and children, different sets of *Red Flags* have been produced for the two populations and we distinguished two different scenarios: the first one with symptoms that are already sufficient to suspect the diagnosis and two additional conditions for children and one for adults that concern associated symptoms in which we recommend instead to investigate the presence of excessive daytime sleepiness and cataplexy, prior referring patients to the reference centers for the diagnosis.

The panel focused particularly in defining the peculiar features of narcolepsy in children and mainly the discrete manifestation of excessive daytime sleepiness, which not always presents with sleep attacks and the typical phenotype of cataplexy in children.

Indeed, the best predictor of a delayed diagnosis, along with the absence of cataplexy, is known to be the pediatric onset of symptoms [26]. This is because pediatric narcolepsy near the onset of the disease may have peculiar features [3, 36], as excessive daytime sleepiness may be manifested as paradoxical hyperactivity, elongation of nighttime sleep, resume of post prandial sleep and symptoms like hallucinations, RBD, and confusional arousals may be difficult to be articulated by children [26, 27]. Similarly, cataplexy at onset may present with the peculiar subcontinuous hypotonia and “cataplectic face” with superimposed dyskinesia or motor hyperactivity resembling other neurodevelopmental disorders [23, 24, 28].

Experts also focused on the description of symptoms different from excessive daytime sleepiness and cataplexy, since both the AWAKEN survey [21] and our own survey highlighted that clinicians may be more confident in investigating the cardinal features of narcolepsy (i.e., excessive daytime sleepiness and cataplexy), while less so in identifying the remaining symptoms typical of the narcolepsy pentad, i.e., hallucinations, sleep paralysis, and disrupted nocturnal sleep with RBD.

This report, by producing *Red Flags* guiding in suspecting the diagnosis of narcolepsy, has been therefore an attempt to overcome the major barriers identified through the survey.

## Electronic supplementary material

ESM 1 (PDF 48.9 mb)
