# Supplementary material for: Red Flags for early referral of people with symptoms suggestive of narcolepsy: a report from a national multidisciplinary panel
Source: Neurol Sci. 2018 Dec 12;40(3):447–56. doi: 10.1007/s10072-018-3666-x (PMC6433801; doi:10.1007/s10072-018-3666-x)
Supplement: Supplementary file 1 — (PDF 48.9 mb) [file 10072_2018_3666_MOESM1_ESM.pdf]

***Supplementary material* - Neurological Sciences**

Red Flags for early referral of people with symptoms suggestive of narcolepsy: a report from a national multidisciplinary panel

Vignatelli L , Antelmi E, Ceretelli I , Bellini M , Carta C , Cortelli P, Ferini-Strambi L, Ferri R, Guerrini R, Ingravallo F, Marchiani V, Mari F, Pieroni G, Pizza F, Verga MC, Verrillo E, Taruscio D, Plazzi G

Corresponding author: Giuseppe Plazzi, Department of Biomedical and Neuromotor Sciences (DIBINEM), University of Bologna, via Ugo Foscolo n 7, 40123, Bologna, Italy. Fax: +39 051 4966176. E-mail address: giuseppe.plazzi@unibo.it. ORCID 0000-0002-1051-0472

Red flags per il paziente pediatrico

Sospettare la narcolessia nel(la) bambino/a che giunga a visita per il manifestarsi di …

1. **… eccessiva sonnolenza diurna e/o cataplessia** (vedi Box 1a e Box 1b per il dettaglio).
   →*In questo caso inviare ad indagine presso centro specializzato in medicina del sonno.*

2. … **sintomi atipici nel sonno** (**allucinazioni ipnagogiche/ipnopompiche, paralisi del sonno, sonno notturno disturbato)** (vedi Box 2 per il dettaglio).
   →*In questo caso indagare l'eventuale presenza di eccessiva sonnolenza diurna o cataplessia (Red Flag 1), anche se non spontaneamente riportate. Nel caso uno di questi sintomi sia sospettato, inviare ad indagine presso centro specializzato in medicina del sonno.*

3. … segni di **pubertà precoce** o incremento ponderale fino alla comparsa di **obesità** (vedi Box 3 per il dettaglio**).**
   → *In questo caso indagare l'eventuale presenza di eccessiva sonnolenza diurna o cataplessia (Red Flag 1), anche se non spontaneamente riportate. Nel caso uno di questi sintomi sia sospettato, inviare ad indagine presso centro specializzato in medicina del sonno.*

BOX 1a. Dettaglio della *Red Flag* 1 paziente pediatrico sulla eccessiva sonnolenza diurna. Questa *Red Flag* è stata formulata in base alla seguente letteratura: criteri diagnostici internazionali (# 1), una *evidence-based clinical summary* (# 9), tre revisioni (# 3, 11, 13), uno studio di coorte (# 5), uno studio trasversale di incidenza (# 12), un *case report* (# 14).

Nel paziente pediatrico l'**eccessiva sonnolenza diurna** può manifestarsi in uno dei seguenti modi

- … **addormentamenti improvvisi**, in situazioni inusuali-non monotone, spesso di breve durata, con effetto ristoratore e, spesso, accompagnati da sogni vividi.
- ... **modificazione (acuta o progressiva) del ritmo sonno veglia** con la tendenza a prolungare le ore totali di sonno notturno, spesso con aumento del numero dei risvegli, oppure con la ripresa dell'abitudine a fare il pisolino pomeridiano (in genere dopo i 5-6 anni di età) o la tendenza ad andare a letto prima alla sera o a svegliarsi più tardi e con più difficoltà al mattino.
- … comparsa durante la giornata di **iperattività, disattenzione, irritabilità e comportamenti automatici.** Tali sintomi sono particolarmente osservabili a scuola, ad esempio scrivere con una grafia grossolana, o con parole improprie fino alla completa interruzione della scrittura.

Ricorda: nei bambini talvolta la narcolessia viene confusa con privazione cronica di sonno, disagio psicologico, crisi epilettiche, eventi infettivi. Pertanto nel caso si sospettasse una di queste condizioni considerare anche la narcolessia tra le diagnosi differenziali.

BOX 1b. Dettaglio della *Red Flag* 1 paziente pediatrico sulla cataplessia. Questa *Red Flag* è stata formulata in base alla seguente letteratura: criteri diagnostici internazionali (# 1), una *evidence-based clinical summary* (# 9), una revisione (# 3), uno studio di coorte (# 5), due serie di casi (# 4, 7), due *case-reports* (# 15, 16).

Nel paziente pediatrico la **cataplessia** può manifestarsi in uno dei seguenti modi

- … episodi di **perdita di tono muscolare** in assenza di perdita di coscienza, scatenati da emozioni, solitamente brevi (secondi-minuti). La perdita di tono può essere parziale (ptosi palpebrale, spianamento della rima buccale, ptosi della bocca, caduta del capo, modificazione improvvisa e transitoria dell'eloquio, con abbassamento del tono della voce e parola come da ubriaco), e/o generalizzata (caduta a terra).
- … **facies ipotonica** con tendenza subcontinua e fluttuante in intensità ad avere la mandibola semi-aperta, la lingua protrusa, gli occhi semichiusi. La facies ipotonica può essere un fenomeno subcontinuo con esacerbazioni intermittenti, correlate o meno alle emozioni o alle attività della vita quotidiana (mangiare, giocare, fare un compito che richiede un coinvolgimento emotivo quale un video-game, film. ecc).
- … **aspetto generale ipotonico**, che può essere diffuso al capo-collo (con episodi di caduta/iperestensione del capo), al tronco (con episodi di flessione del tronco) o a tutto il corpo e gli arti inferiori dando l'impressione di una marcia instabile, che a tratti può simulare una marca atassica.
- … **movimenti attivi** intermittenti, che possono o coinvolgere il volto (smorfie facciali, discinesie peribuccali, tics), o parti del corpo (movimenti simil-coreici, stereotipie con arti superiori o anche del tronco).

Ricorda: tale presentazione entrerà dunque in diagnosi differenziale con malattie neuromuscolari o disturbi del movimento ipercinetici, e tuttavia la presenza di fluttuazioni (non esercizio-indotte) e l'eventuale associazione con eccessiva sonnolenza diurna potranno fungere da supporto anamnestico.

BOX 2. Dettaglio della *Red Flag* 2 paziente pediatrico sui sintomi atipici del sonno. Questa *Red Flag* è stata formulata in base alla seguente letteratura: criteri diagnostici internazionali (# 1), una *evidence-based clinical summary* (# 9), due revisioni (# 3, 20), quattro serie di casi (#6, 17-19, 21).

Nel paziente pediatrico i **sintomi atipici del sonno** possono manifestarsi come

- Fenomeni di **allucinazioni** multimodali (per lo più visive) o illusioni percettive all'addormentamento (ipnagogiche) o al risveglio (ipnopompiche).
- **Sogni** vividi, molto spesso a contenuto terrifico.
- **Paralisi del sonno**, cioè risvegli con l'angosciante sensazione di non riuscire a muoversi (fino ad 1-2 minuti di durata), associati o meno ad allucinazioni/illusioni.
- **Sonno notturno molto disturbato**, con la tendenza ad agitarsi molto nel sonno e/o presenza di **disturbo del comportamento in sonno REM (RBD)** e cioè la presenza durante il sonno di comportamenti motori congrui con il contenuto del sogno (il paziente mima il sogno che sta esperendo e al risveglio spesso racconta/ricorda un sogno vivido).

Ricorda: quando la malattia è conclamata, questi sintomi saranno associati alla ipersonnia o alla cataplessia. Tuttavia, per quanto raro, può accadere che tali sintomi siano il sintomo di esordio. In tal caso, possono essere difficili da indagare per la difficoltà da parte del bambino nel riconoscere e dunque nel raccontare tali sintomi (per scarsa consapevolezza o senso di vergogna).

BOX 3. Dettaglio della *Red Flag* 3 paziente pediatrico sui segni endocrinologici. Questa *Red Flag* è stata formulata in base alla seguente letteratura: criteri diagnostici internazionali (# 1), una *evidence-based clinical summary* (# 9), una revisione (# 3), uno studio di coorte con controlli (# 24), uno studio di coorte retrospettivo (# 25), uno studio trasversale con controlli (# 23), una serie di casi (# 20).

Nel paziente pediatrico la narcolessia può manifestarsi all'esordio anche con **segni endocrinologici** quali la pubertà precoce o l'incremento ponderale improvviso e rapido fino alla comparsa di obesità. Quindi il bambino che manifesti tali quadri, potrebbe avere la narcolessia e, pertanto, sarà opportuno indagare l'eventuale presenza dei sintomi segnalati nelle *Red Flags* 1 e 2.

Red flags per il paziente adulto

Sospettare la narcolessia nell'adulto che giunga a visita per il manifestarsi di …

1. **… eccessiva sonnolenza diurna e/o cataplessia** (vedi Box 4a e Box 4b per il dettaglio).
   →*In questo caso inviare ad indagine presso centro specializzato in medicina del sonno.*

2. … **sintomi atipici nel sonno** (**allucinazioni ipnagogiche/ipnopompiche, paralisi del sonno, sonno notturno disturbato** - vedi Box 5 per il dettaglio).
   →*In questo caso indagare l'eventuale presenza di eccessiva sonnolenza diurna o cataplessia (Red Flag 1), anche se non spontaneamente riportate. Nel caso uno di questi sintomi sia sospettato, inviare ad indagine presso centro specializzato in medicina del sonno.*

BOX 4a. Dettaglio della *Red Flag* 1 paziente adulto sulla eccessiva sonnolenza diurna. Questa *Red Flag* è stata formulata in base alla seguente letteratura: criteri diagnostici internazionali (# 1), due *evidence-based clinical summaries* (# 8, 10), una revisione (# 2).

Nell'adulto l'**eccessiva sonnolenza diurna** può manifestarsi nei seguenti modi

- … **addormentamenti improvvisi**, in situazioni inusuali-non monotone, spesso di breve durata, con effetto ristoratore e accompagnati da sogni vividi, talora associati a comportamenti motoria.
- … **modificazione (acuta o progressiva) del ritmo sonno veglia** con la tendenza a prolungare le ore di sonno nel corso della giornata o avere addormentamenti in situazioni monotone.

Ricorda: nel paziente adulto le diagnosi differenziali più comuni sono sindrome delle apnee ostruttive in sonno, privazione cronica di sonno, disagio psicologico, crisi epilettiche. Pertanto nel caso si sospettasse una di queste condizioni considerare anche la narcolessia tra le diagnosi differenziali.

BOX 4b. Dettaglio della *Red Flag* 1 paziente adulto sulla cataplessia. Questa *Red Flag* è stata formulata in base alla seguente letteratura: criteri diagnostici internazionali (# 1), due *evidence-based clinical summaries* (# 8, 10), una revisione (# 2), tre serie di casi (# 26-28).

Nell'adulto la **cataplessia** può manifestarsi in uno dei seguenti modi

- … episodi di **perdita di tono muscolare** in assenza di perdita di coscienza, scatenati da emozioni, solitamente brevi (secondi-minuti). La perdita di tono può essere parziale (ptosi palpebrale, spianamento della rima buccale, ptosi della bocca, caduta del capo, modificazione improvvisa e transitoria dell'eloquio, con abbassamento del tono della voce e parola come da ubriaco), e/o generalizzata (caduta a terra).
- … episodi caratterizzati da **sospensione dell'eloquio e dell'attività in corso**, in assenza di una chiara ipotonia, sempre con coscienza integra.

Ricorda: nel paziente adulto le diagnosi differenziale più comune è quella delle crisi comiziali ed eventi ischemici transitori. Qualora non sussistano dati anamnestici a favore di questa diagnosi (sospensione del contatto, aura, etc) e in presenza di associazione con eccessiva sonnolenza diurna, la cataplessia andrà sospettata.

BOX 5. Dettaglio della *Red Flag* 2 paziente adulto sui sintomi atipici del sonno. Questa *Red Flag* è stata formulata in base alla seguente letteratura: criteri diagnostici internazionali (# 1), due *evidence-based clinical summaries* (# 8, 10), tre revisioni (#2, 29, 32), due serie di casi (# 30, 31).

Nell'adulto i **sintomi atipici del sonno** possono manifestarsi come

- Fenomeni di **allucinazioni** multimodali (per lo più visive o uditive), illusioni percettive, ipnagogiche (all'addormentamento) o ipnopompiche (al risveglio).
- **Paralisi del sonno,** cioè risvegli con l'angosciante sensazione di non riuscire a muoversi (fino ad 1-2 minuti di durata), associati o meno ad allucinazioni/illusioni.
- **Sonno notturno molto disturbato**, con sogni vividi e la tendenza ad agitarsi molto del sonno e/o ad agire i propri sogni configurando dunque un **disturbo del comportamento in sonno REM (RBD)** e cioè la presenza durante il sonno di comportamenti motori congrui con il contenuto del sogno (il paziente mima il sogno che sta esperendo e al risveglio spesso racconta/ricorda un sogno vivido).

Ricorda: se questi fenomeni sono isolati e non associati ad ipersonnia e/o cataplessia, il più delle volte sono situazioni parafisiologiche o nel caso dell'RBD può trattarsi di una condizione isolata (idiopatica) o del prodromo di una malattia neurodegenerativa (malattia di Parkinson, demenza a corpi di Levy, atrofia multisistemica). In presenza di uno o più di questi fenomeni invece, è bene indagare la presenza di eccessiva sonnolenza diurna ed episodi suggestivi di cataplessia.

Bibliografia

1. American Academy of Sleep Medicine. *The International Classification of Sleep Disorders: Diagnostic & Coding Manual*. 2nd ed. Rochester, MN: American Academy of Sleep Medicine; 2005
2. Scammell TE (2015) Narcolepsy. N Engl J Med 373:2654-2662.
3. Postiglione E, Antelmi E, Pizza F, et al (2018) The clinical spectrum of childhood narcolepsy. Sleep Med Rev 38:70-85
4. Plazzi G, Pizza F, Palaia V, et al (2011) Complex movement disorders at disease onset in childhood narcolepsy with cataplexy. Brain 134:3477-3489.
5. Pizza F, Franceschini C, Peltola H, et al (2013) Clinical and polysomnographic course of childhood narcolepsy with cataplexy. Brain 136:3787-3795.
6. Dunne L, Patel P, Maschauer EL, Morrison I, Riha RL (2016) Misdiagnosis of narcolepsy. Sleep Breath 20:1277-1284.
7. Serra L, Montagna P, Mignot E, Lugaresi E, Plazzi G (2008) Cataplexy features in childhood narcolepsy. Mov Disord 23:858–865.
8. DynaMed Plus [Internet]. Ipswich (MA): EBSCO Information Services. 1995 - . Record No. 116132, Narcolepsy; [updated 2018 Mar 09, cited place cited date here]; [about 29 screens]. Available from http://www.dynamed.com/login.aspx?direct=true&site=DynaMed&id=116132. Registration and login required. Last access May 28, 2018.
9. Kotagal S. Narcolepsy in children. Scammell TE, Chervin RD, Eichler AF, eds. UpToDate. Waltham, MA: UpToDate Inc. http://www.uptodate.com. Last access May 28, 2018.
10. Scammell TE. Clinical features and diagnosis of narcolepsy in adults. Benca R, Eichler AF, eds. UpToDate. Waltham, MA: UpToDate Inc. http://www.uptodate.com. Last access May 28, 2018.
11. Wise J, Lynch J (2001) Narcolepsy in children. Semin Pediatric Neurol 8:198-206.
12. Partinen M, Saarenpää-Heikkilä O, IlveskoskIi, et al (2012) Increased incidence and clinical picture of childhood narcolepsy following the 2009 H1N1 pandemic vaccination campaign in Finland. Plos One 2:e33723.
13. Babiker MO, Prasad M (2015) Narcolepsy in children: a diagnostic and management approach. Pediatr Neurol 52:557.
14. Lee J, Na G, Joo EY, et al (2017) Clinical and polysomnographic characteristics of excessive daytime sleepiness in children. Sleep Breath 21:967.
15. Prasad M, Setty G, Ponnusamy A, et al (2014) Cataplectic facies: clinical marker in the diagnosis of childhood narcolepsy-report of two cases. Pediatr Neurol 515:517.
16. Dhondt K, Verhelst H, Pevernagie D, Slap F, Van Coster R (2009) Childhood narcolepsy with partial facial cataplexy: a diagnostic dilemma. Sleep Med 10: 797-798.

17. Nevsimalova S, Prihodova I, Kemlink D, et al (2007) REM behavior disorder (RBD) can be one of the first symptoms of childhood narcolepsy. Sleep Med 8:784.
18. Lloyd R, Tippmann-Peikert M, Slocumb N, Kotagal S (2012) Characteristics of REM sleep behavior disorder in childhood. J Clin Sleep Med 8:127.
19. Pisko J, Pastorek L, Buskova J, Sonka K, Nevsimalova S (2014) Nightmares in narcolepsy: underinvestigated symptoms? Sleep Med 15:967-972
20. Jardri R, Bartels-Velthuis, Debbané M, et al (2014) From phenomenology to neurophysiological understanding of hallucinations in children and adolescents. Schizophr Bull 40:S221-S232
21. Antelmi E, Pizza F, Vandi S, et al (2017) The spectrum of REM sleep-related episodes in children with type 1 narcolepsy. Brain 140:1669-1679.
22. Dahmen N, Bierbrauer J, Kasten M (2001) Increased prevalence of obesity in narcoleptic patients and relatives. Eur Arch Psychiatry Clin Neurosci 251:85.
23. Poli F, Pizza F, Mignot E, et al (2013) High prevalence of precocious puberty and obesity in childhood narcolepsy with cataplexy. Sleep 36:175.
24. Wang Z, Wu H, Stone WS, et al (2016). Body weight and basal metabolic rate in childhood narcolepsy: a longitudinal study. Sleep Med 25:139.
25. Ponziani V, Gennari M, Pizza F, et al (2016) Growing Up with Type 1 Narcolepsy: Its Anthropometric and Endocrine Features. J Clin Sleep Med 12:1649.
26. Vetrugno R, D'Angelo R, Moghadam KK, et l (2010) Behavioural and neurophysiological correlates of human cataplexy: a video-polygraphic study. Clin Neurophysiol 121:153-162.
27. Overeem S, van Nues SJ, van der Zande WL, et al (2011) The clinical features of cataplexy: a questionnaire study in narcolepsy patients with and without hypocretin-1 deficiency. Sleep Med 12:12
28. Pizza F, Antelmi E, Vandi S, et al (2018) The distinguishing motor features of cataplexy: a study from video-recorded attacks. Sleep 41(5). doi: 10.1093/sleep/zsy026.
29. Plazzi G, Serra L, Ferri R (2008) Nocturnal aspects of narcolepsy with cataplexy. Sleep Med Rev 12: 109-128
30. Franceschini C, Ferri R, Pizza F, et al (2011) Motor events during REM sleep in patients with narcolepsy-cataplexy: a video-polysomnographic pilot study. Sleep Med 12:S59-63.
31. Cipolli C, Franceschini C, Mattarozzi K, et al (2011) Overnight distribution and motor characteristics of REM sleep behaviour disorder episodes in patients with narcolepsy-cataplexy. Sleep Med 12:635.
32. Dauvilliers Y, Jennum P, Plazzi G (2013) Rapid eye movement sleep behavior disorder and rapid eye movement sleep without atonia in narcolepsy. Sleep Med 14:775-781
